# Genetic Variability Trend of Lusitano Horse Breed Reared in Italy

**DOI:** 10.3390/ani12010098

**Published:** 2022-01-01

**Authors:** Maria Cristina Cozzi, Paolo Valiati, Maria Longeri, Carlos Ferreira, Sofia Abreu Ferreira

**Affiliations:** 1 Dipartimento di Medicina Veterinaria, Università degli Studi di Milano, Via dell’Università, 6-26900 Lodi, Italy; paolo.valiati@unimi.it (P.V.); maria.longeri@unimi.it (M.L.); 2 Laboratório de Genética Molecular de Alter, Instituto National de Investigação Agraria e Veterinaria (INIAV, IP), Unidade Estratégica de Investigação e Serviços de Biotecnologia e Recursos Genéticos, Coudelaria de Alter, 7440-152 Alter do Chão, Portugal; carlos.ferreira@iniav.pt (C.F.); sofia.ferreira@iniav.pt (S.A.F.)

**Keywords:** Lusitano Horse, Italy, genetic variability, microsatellite markers, allele frequencies, inbreeding

## Abstract

**Simple Summary:**

The Lusitano Horse (LH) originates from Portugal, but is reared worldwide. Since 1994, the University of Milan has tested the LHs breed reared in Italy for parentage control using microsatellite markers. This study aims to assess the genetic variability of the LHs reared in Italy for more than four decades, in order to obtain information necessary for genetic management.

**Abstract:**

The Lusitano Horse (LH) originates from Portugal, but is reared worldwide. Since 1994, the University of Milan has routinely tested the LHs bred in Italy for parentage control. This study aims to assess the genetic variability of the LH reared in Italy using 16 microsatellites markers. Moreover, the genetic variability changes over the years in the total population (n.384) and in unrelated horses (n.47) were evaluated. Horses were grouped according to their date of birth (1975–1990, 1991–2000, 2001–2010, 2010–2019). Standard genetic diversity parameters, including observed (Ho) and expected (He) heterozygosity, Hardy-Weinberg equilibrium (HWE; P-Val), allelic richness, and inbreeding coefficient (F_is_) were estimated. In the whole period, the total population showed Ho as high as 0.69, low F_is_ (0.057), and imbalance for HWE. When considering the unrelated horses, Ho was seen to increase over time (from 0.594 in 1975–1990 to 0.68 in 2010–2019) and frequencies were in HWE, again having low and decreasing values of Fis (from 0.208 in 1975–1990 to 0.019 in 2010–2019). Bottleneck analysis excluded a recent population decline. Principal Coordinate Analysis at the individual level defined two clusters, the major cluster including all the most recent horses. An increasing number of dams (156% more from 2001–2010 to 2011–2019) supports the good variability recorded in the population so far. However, the high number of foals (77.2%) sired by only four stallions in recent years suggests caution in the choice of the sires for the future.

## 1. Introduction

The Lusitano Horse (LH) is one of the most ancient equine breeds. It originates from Portugal, where it is economically the most important horse breed, with a registered population of about 5000 mares and 1000 stallions [1,2]. Since being established in 1967, the main official studbook of the LH in Portugal is managed by the Associação Portuguesa de Criadores do Cavalo Puro Sangue Lusitano (ASPL) who keeps records of birth, pedigree, and morphological data of the LH bred worldwide. Indeed, the breed is reared in many countries, with Brazil recording the second largest population [2], followed by France, and then Spain [1]. All the Lusitano sires registered in the official studbook as breeding stock must be submitted to a morpho-functional test and scored on gaits and conformation. The Lusitano mares are scored when they are presented by hand to a jury of experts [1]. Sires enter reproduction at 7 years and mares at 5.5 years on average. Indeed, sires are trained and used in equestrian activities for several years, while mares are frequently used for breeding only. This implies that horses delay their reproductive career, with a rather long generation interval, such as other saddle breeds [1,3]. The long reproductive career of breeding individuals leads to the possibility of an increase in inbreeding. For this reason, the genetic variability must be carefully monitored.

In Italy, the Associazione Italiana Cavallo Puro Sangue Lusitano (AICL) was established in 2010 to promote the breed and its selection. A reciprocal partnership between the APSL and the AICL was signed in the same year. The agreement provides for all LHs born in Italy to be enrolled in the Portuguese studbook, after parentage verification.

Currently, approximately 500 LHs are reared by 15 breeders in Italy and they are mainly trained as riding horses but are also used for other equestrian disciplines, such as carriage driving, working equitation, and dressage [4]. Since 1994, the Laboratory of Animal Genetics and Genomics at the University of Milan (Italy) has performed parentage tests for the breed, initially by blood groups and protein polymorphisms, then by microsatellite (STR) markers, always following the International Society of Animal Genetics (ISAG) guidelines. Specimens were maintained at the University’s repository (Animal Bio Arkivi) and 433 are available for the period 1994–2019.

The genetic variability of the LH using pedigree data and STR markers has been described in several studies [1,2,3,5,6]. STR markers have been widely used to analyze the genetic diversity and population structure of other both cosmopolitan and local horse breeds [7,8,9,10,11,12,13]. STRs are used worldwide for routine equine genotyping applied to forensics, parentage, and kinship analysis [14,15].

The present study on 384 samples collected over the years in Italy and typed for a panel of STR markers aimed to analyze the genetic variability (i) on the 1975–2019 period considering the population as a whole and (ii) on the animals grouped in four subpopulations according to the decade of birth in order to verify the changes in genetic variability that occurred in this time.

## 2. Materials and Methods

### 2.1. Samples and Laboratory Methods

The Laboratory of Animal Genetics and Genomics at the University of Milan (Italy) maintains the blood specimens of all the LHs bred in Italy (433 horses), collected over the years (1994 to 2019), in its bio-repository (Animal Bio Arkivi). Specimens consist of EDTA whole peripheral blood kept at −20 °C. All applicable international, national and/or institutional guidelines for the care and use of animals were followed. The stored samples were approved by the Ethics Committee of Università degli Studi di Milano Prot. OPBA_56_2016.

Out of the LH stored, 384 (165 males and 219 females) were requested to be typed for parentage control in the period 1994–2019. Genomic DNA was extracted from EDTA blood samples using a commercial kit (Zymo Quick-gDNA MicroPrep). DNA samples were genotyped using 17 STR markers of StockMarks for the Horses^®^ Genotyping Kit, (AHT4, AHT5, ASB2, ASB17, ASB23, CA425, HMS1, HMS2, HMS3, HMS6, HMS7, HTG4, HTG6, HTG7, HTG10, LEX3, and VHL20). The commercial panel included the 12 STRs of the official ISAG panel for parentage testing (AHT4, AHT5, ASB2, ASB17, ASB23, HMS2, HMS3, HMS6, HMS7, HTG4, HTG10, and VHL20) (http://www.isag.us/Docs/EquineGenParentage2016.pdf, accessed on 30 November 2021) [16]. PCR protocol followed the manufacturer’s instructions [17]. The PCR products were run on an ABI PRISM^®^ 310 Genetic Analyzer and fragments were analyzed by GeneScan^®^ and Genotyper^®^ software. Genotypes at the same loci of 87 LHs (26 males and 61 females), parents of foals born in Italy and tested at Laboratório de Genética Molecular de Alter (PT), were also available. The ChrX marker LEX03 was excluded by the statistical analyses.

Out of the 384 genotyped horses, the analyses were performed on the following animal groups and periods, the generation interval periods defined according to Vicente 2015 [3] (Table 1). 

### 2.2. Genetic Diversity Measures

Allelic frequencies and Hardy-Weinberg equilibrium (HWE; P-Val) were estimated using the GENEPOP v. 7 software [18,19]. The P-Val of the exact HWE tests for all locus/population combinations were obtained using Markov chain permutations with 10,000 dememorizations and 400 batches, 10,000 iterations each. 

The observed number of alleles (Na) and the observed mean number of alleles value (MNa), the effective number of alleles (Ae) and the mean effective number of allele value (MAe), the Nei’s expected heterozygosity (N_He), and the observed (Ho) and expected (He) heterozygosity per locus and for all were calculated with POPGENE v.1.32 software [20].

Allelic richness (Ar) per locus and in the population (mean allelic richness MAr) was standardized for variations in sample size and was calculated using FSTAT v. 2.9.3 [21].

The number of private alleles per population (N. PrA), the number of alleles with a frequency >5% (Na Freq. > 5%), and the unbiased expected heterozygosity (uHe) were estimated using GenAlex v. 6.503 [22]. 

### 2.3. Inbreeding Analysis

Inbreeding coefficient (F_is_) was estimated using the GENEPOP v. 7 software [18,19].

The average number of pairwise differences between the groups was calculated using Arlequin v. 3.5.2 software [23].

Bottleneck events were tested in the unrelated horses on the four generation interval groups and in the total population in the same four generation intervals two methods were applied: (1) the “Two excess heterozygosity tests and Wilcoxon sign-rank test” [24]. This method calculates the probability distribution using 1000 simulations under three evolutionary models: the Infinite allele model (IAM), stepwise mutation model (SMM), and two-phase model of mutation (TPM) set with 95% single-step mutational events at 12% variance; (2) the “Graphical representation of the mode-shift indicator” [25]. This method examines distortion on the expected L-shaped distribution of allele frequency and determines if the population is under mutation drift equilibrium (L-shaped curve) or not (mode shift). These two methods were applied using Bottleneck v1.2.02 [26].

### 2.4. Population Structure

FSTAT v. 2.9.3 [21] was used to estimate Wright’s F statistics (F_IT,_ and F_ST_), according to Weir and Cockerham (1984) [27].

The dissimilarity matrix, the Principal Coordinates Analysis (PCoA), and the dispersion graph were performed using GenAlex v. 6.503 [22]. 

## 3. Results

### 3.1. Genetic Diversity

#### 3.1.1. Period 1975–2019

The genetic variability parameters were calculated for each marker in both the total population (related and unrelated, n.384) and the unrelated horses (at least in the first generation, n.47) (Tables S1 and S2 and Figure S1). Among the horses born in the period 1975–2019 and genotyped at 16 STS loci, 132 allelic variants were detected in the total population (mean number of allele/locus 8.2; Table S1) and 118 in the unrelated horses (mean number of allele/locus 7.3; Table S2), respectively. 

**Figure 1:**
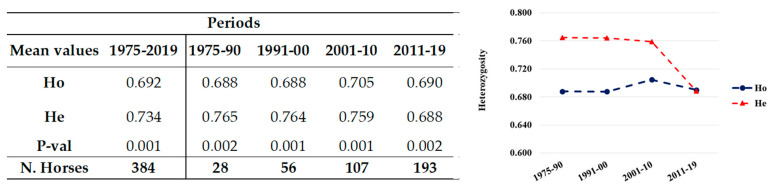
Mean values (**left**) and their plot (**right**) of the observed (Ho) and expected (He) heterozygosity of the total (related and unrelated) population in different periods. The number of horses and the HWE (P-val) per group are reported on the left.

The total population showed significantly different Ho and He (0.692 and 0.734, respectively), resulting to significant deviation from HWE (Mean P-val 0.001; Figure 1), as expected in a population including related individuals, with eight loci being highly significant P-val (Figure 1). However, when only the unrelated horses were considered, the difference between Ho (0.684) and He (0.725) reduced and the population were in HWE (Mean P-val 0.423) with only one locus out of equilibrium (CA425, P-val = 0.033; Table S2).

#### 3.1.2. Periods 1975–1990, 1991–2000, 2001–2010, 2011–2019

**Figure 2:**
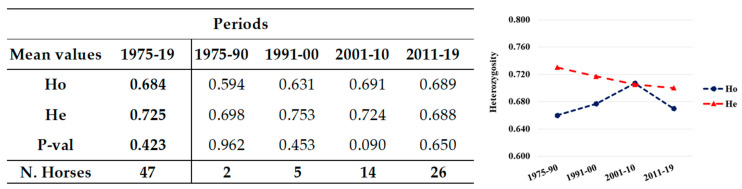
Mean values (**left**) and their plot (**right**) of the observed (Ho) and expected (He) heterozygosity of unrelated horses in different periods. The number of horses and the HWE (P-val) per group are reported on the left.

Both the unrelated and the total population were also grouped for analyses by date of birth into generation groups (1975–1990, 1991–2000, 2001–2010, 2011–2019). The number of horses per group always gradually and progressively increased over time, approximately doubling from one period to the next (Figure 1 and Figure 2).

In the related and unrelated horses, Ho remained stable in time (range 0.688–0.690), constantly lower than He (ranging 0.765–0.688), apart from in the last period where their values came very close. The groups were always to deviation from HWE in each period (P-val < 0.05).

Several STR loci in every group deviated from HWE, HMS1, and HTG7 deviating in every period and HTG6 being in equilibrium only in the first period (1975–1990) (Table S3). Private alleles, defined here as alleles exclusively identified in horses of a single period among the other periods, were identified in each period. The first (1975–1990) and the last (2011–2019) periods showed the highest mean number of private alleles (PrA = 0.5; Figure S2).

Additionally, in the unrelated individuals (Figure 2), He showed a decreasing trend in the last two periods, which was more evident in the period 2011–2019. Overall, Ho had lower values than He, but showed progressive increases, reaching in the last period a value (0.689) as high as He (0.688) and as high as both Ho (0.69) and He (0.688) of the total population in the same period (0.690 vs. 0.688, respectively: Figure 1). Populations were in HWE in all the groups. In these unrelated horses, there were no private alleles, as they were exclusively found in foals of the related group.

### 3.2. Inbreeding Analysis

Overall, F_is_ values were quite low, in accordance with high values of heterozygosity (Table 2). When considering the different periods, F_is_ showed a decrease in time both in the total and in the unrelated population, which is consistent with the increase in Ho in the unrelated population previously reported (F_is_ values in the periods 1975–1990 and 1991–2000 are affected by the extreme low sample numbers). This sign of low inbreeding could be ascribed to the population structure regarding the number of dams and sire.

**Figure 3:**
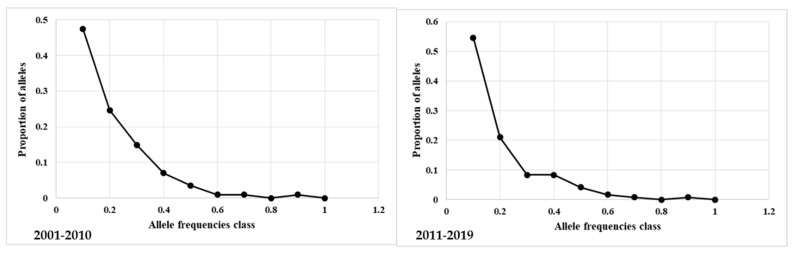
Expected numbers of loci with heterozygosity excess by Bottleneck software. In the plot “L” shaped distribution of allelic class vs proportion of alleles in related and unrelated LH, periods 2001–2010 and 2011–2019.

The bottleneck test was applied to the total and unrelated animals in the four generation interval groups. The expected numbers of loci with heterozygosity excess for the three evolutionary models, the non-parametric Wilcoxon rank test, and the probability values calculated for the three models were reported in Table S4. The plot of allelic class vs. proportion of alleles showed a normal “L” shaped distribution in all groups (Figure 3 and Figure S3). The L-shaped curve indicates the abundance of alleles with low frequency (<0.10). The test results indicate that, due to mutation-drift equilibrium, the total and unrelated horses in the four populations did not undergo a recent genetic bottleneck.

### 3.3. Population Structure

The inbreeding estimate of an individual relative to the total population (F_IT_) showed values as low as 0.041 in the total population (n.384) and 0.064 in the unrelated horses (n. 47). Additionally, the proportion of genetic variance contained in the subpopulations relative to the total population variance (F_ST_) were in low values (F_ST_ = 0.027) in the total population and very low values (F_ST_ = 0.019) in the unrelated horses. The F_ST_ analysis performed on the total population showed the highest diversity within the second-generation interval group (1991–2000; πX = 6.831), and the lowest in the third group (2011–2019; πX = 6.509). The last group (2011–2019) showed the highest differences with the previous ones when assessing the pairwise differences between populations (2011–2019 vs. 1975–1990: πXY = 6.811; 2011–2019 vs. 1991–2000: πXY = 6.850; 2011–2019 vs. 2001–2010: πXY = 6.784), which is consistent with the results of the corrected average pairwise difference (below, values on the gray diagonal). Again, according to the chi-square test and permutation test of F_ST_, the group 2011–2019 showed significant divergence from the others (*p* < 0.05, Table 3).

**Figure 4:**
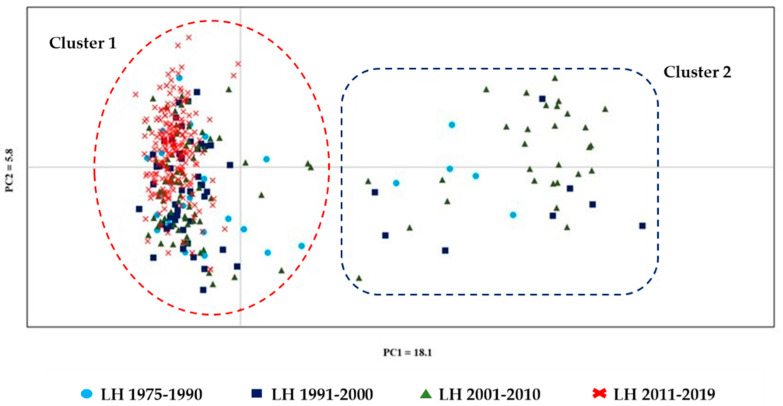
Graphical dispersion of inter-population distances of the LH bred in Italy. The Cartesian axes obtained by the Principal Coordinates Analysis (PCoA) were based on the dissimilarity matrix of 384 related and unrelated horses.

The graphic dispersion of inter-population distances and individual LH in the four generation interval periods, obtained by the Principal Coordinates Analysis (PCoA), is displayed in Figure 4. The PCoA, based on the individual distance matrix of the total 384 horses, showed that the first and second PCs explained 18.1% and 5.8% of the genetic diversity, respectively. Individuals were grouped in two clusters: one included all the individuals belonging to the most recent period (2010–2019) and most of the individuals belonging to the previous periods (Cluster 1—red dashed circle). The second (Cluster 2—blue dashed square) included 17.8% of the horses bred in 1975–1990, 14.2% of those bred in 1991–2000), and 26.2% of those bred in 2001–2010. 

As shown by the parentage tests performed on the STR profiles, the composition of Italian LH breeders (related and unrelated) is reported in Table 4. Overall, the numerosity of the recorded horses was particularly low in the first period because the breed was at its historic beginnings in Italy. It is worth mentioning that the LH breeders were more numerous in the country than reported in the present study, but they were not included because STRs as routine markers were not available at the time and genotyping was largely performed abroad for a while. Progressively the number of LH bred and genotyped in Italy increased.

The number of mares progressively increased over time. In the period 2001–10 out of 32 mares, 84.4% delivered one single or two foals. In the last decade (2011–19), the number of dams more than doubled (156% increase) and the number of mares with only one or two foals decreased but remained high (to 68.3%).

Similarly, males also increased their numerosity in time, but with a lower multiplication factor than mares in the last two periods (51.9% increase from 2001–10 to 2011–19).

**Figure 5:**
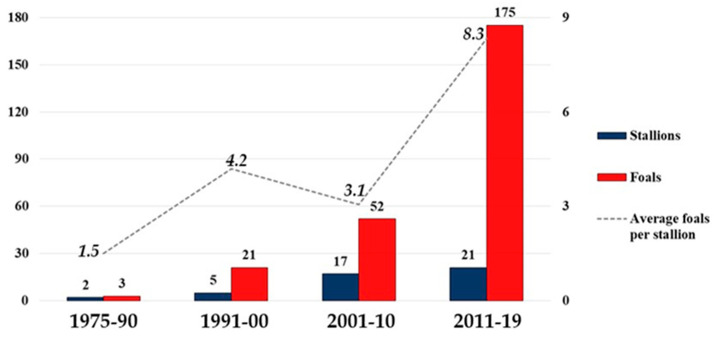
The histogram shows the number of stallions and their offspring (foals) counted in the four generation interval groups 1975–1990; 1991–2000; 2001–2010; 2011–2019 (values on the left axis). The histogram shows sires with ≥2 foals. The dotted line shows the average number of foals/stallion in each group (values on the right axis).

The number of foals consistently increased over time; it was three in 1975–1990, 21 in 1991–2000, 49 in 2001–2010, and 175 in 2011–2019. Additionally, an increase in the average number of foals per stallion was recorded. It started from 1.5 in 1975–1990 and reached an average of 8.3 in 2011–2019 (dotted line, Figure 5). The histograms in Figure 5 show the sires with ≥2 foals. 

In the last period, 2011–2019, four stallions sired a large part of the offspring (approximately 77.2%). Despite the increased number of mothers of foals in this period, 83.1% of the full sibs belonged to only three sires (Table 5).

## 4. Discussion

Management of genetic variability in domestic animals is a priority and it is essential in breeding programs. Changes in the genetic variability of a population over time can be assessed by evaluating the degree of inbreeding. In the present study, the picture of the genetic variability in LH bred in Italy was assessed using 16 microsatellite markers and analyzing the horses bred in the period 1975–2019. To evaluate the breed changes over time, analyses were also carried out that categorized the horses into four groups according to the date of birth (1975–1990, 1991–2000, 2001–2010, and 2011–2019). Moreover, both in the whole time and in the four periods, the total population (related and unrelated horses) or only the unrelated individuals were considered in turn.

The first two decades showed a low number of genotyped horses, due to the small size of the Italian population at the time and the scarce availability of STR profiles, due to those markers having been introduced only from the mid-1990s onwards in horse parentage control routines. Thus, horses bred in the 1970s and 1980s were genotyped later with STRs when requested and mainly abroad. LH is an autochthonous Portuguese breed. Italian breeders started importing individuals for breeding between the middle 1980s and 1990s and gradually started to use mainly local breeding stock. General variability parameter values, recorded in the examined Italian total population (such as the mean number of allele, MNa = 7.375; Table S1), resulted in comparable values to those recorded in countries where LH is more widely bred: Spain (MNa = 7.75), Brazil (8.88), and Mexico (MNa = 8.25) (Table S5) [3]. Additionally, the overall F_IS_ value (0.057) was similar to the values obtained in the LH bred in France (0.040), Brazil (0.038), and Spain (0.042) [3,9] (Table S5).

The Ho was quite constant over time in the total population. Despite a decrease in He (He = 0.688) that slightly exceeded the Ho value (0.69) in 2011–2019, a higher He in allele distribution always drove the populations in all the time groups always out of HWE in each period (P-val < 0.05), which is consistent with the same individuals in the whole period 1975–2019 (Figure 1).

Moreover, in unrelated horses, a very small decrease in He in the last period was recorded, but Ho was constantly increasing with F_is_ low or decreasing over time. The populations were always in HWE, as can be expected by a “not real” population, which is not subject to selection. Good heterozygosity was confirmed by the low values of F_is_, which was particularly low in the last period (2010–2019), in accordance with the high values of Ho (Table 2).

Despite the low values of inbreeding parameters and only a small downward trend of He and Ho in the total population and He in the unrelated population, to fully exclude a bottleneck event, an analysis was performed with software Bottleneck v1.2.02. All the results obtained both in the unrelated horses in the four generation interval groups and related groups confirmed the absence of bottleneck events in the recent history of the Italian LH (Figure 3; Table S4 and Figure S3).

The PCoA analysis based on the individual distance matrix showed a cluster (Cluster 1—red dashed line) including individuals from all the periods considered. This cluster included all the dams and sires of horses of the last decade (Figure 3). Data on the consistency of breeding stock and offspring showed an evident increase in mares as mothers of foals in the last period (156%), but not of sires, at least not with the same magnitude (51.9%; Table 4). The number of foals/sires that strongly increased in recent decades and the high number of half and full sibs was partially counteracted by the high number of different dams the stallions mated (Table 5), resulting in values of Ho as high as 0.69 (Figure 1) and very low inbreeding coefficients (Table 1). However, the PCoA well described the present Italian LH population, in which the low number of stallions and the extremely low number of them (three-four) that generate more than 75% of the foals is a potential premise for a possible increased risk of inbreeding. 

The low number of stallions used by breeders in all periods of analysis, and especially so in the last ten years (Figure 5), had an impact on the allele frequencies, as shown in the total population by the deviation from HWE of some STRs (Table S1). In this regard, STR HTG7 is worthy of attention. A low number of alleles in HTG7 (four to six alleles) was recorded in local populations as Akhal-Teke [12,28], Lithuanian Horse [7], Polish Konig Horse [29], Colonial Spanish Horse [30], and Sanfratellano Horse [8] and in a cosmopolitan breed such as Thoroughbred (four alleles) [13,31]. Additionally, in the present study, HTG7 showed a low number of alleles (from a minimum of four to a maximum of six in the different groups) and a decrease in frequencies of some alleles along the time from the first (1975–90) to the last period (2010–19). In the last period, the alleles identified were six, but two were private and rare (HTG7/P 0.08% and HTG7/Q 0.05%), a consequence of the introduction of two mares (Table S6). HTG7/L identified since the first period (3.7%) decreased to the last period, where it was missing, similarly to what was described by Glazewska et al., 2018 [32] in Polish Arabian horses because of breeding selection.

## 5. Conclusions

This study evaluated the genetic variability of the LH bred in Italy. The population recorded a good genetic variability with a low degree of inbreeding. Indeed, STR markers showed a level of heterozygosity comparable with those found in the LH populations reared in other countries. Nevertheless, considering all the horses, related and unrelated (all potentially breeders), a small trend towards some decrease in heterozygosity in the last ten years was recorded which was not strong enough to generate concern at this moment in time. The observed trend, however, suggests the need for careful genetic management of the population in the coming years, and more emphasis on the use of an increased number of different males as sires.

## Figures and Tables

**Table 1:** Number of samples and generation interval periods.

Horses	Total Period	Generation Interval Periods			
	1975–2019	1975–1990	1991–2000	2001–2010	2010–2019
Total population: Related and unrelated	384	28	56	107	193
Unrelated	47	2	5	14	26

**Table 2:** Mean F_is_, values in the total population (related and unrelated) and the unrelated horses in different periods.

	Mean Value	Periods				
		1975–2019	1975–1990	1991–2000	2001–2010	2011–2019
Total	F_is_	0.057	0.102	0.101	0.072	−0.002
Unrelated	F_is_	0.056	0.208	0.206	0.045	0.019

**Table 3:** Average pairwise differences between each generation interval period in the total population (related and unrelated horses). Above the diagonal (blue): average number of pairwise differences between populations (πXY); Diagonal elements (gray): average number of pairwise differences within the population (πX); Below diagonal (white): corrected average pairwise difference (πXY − (πX + πY). * = significant difference between the groups (*p* < 0.05).

	1975–1990	1991–2000	2001–2010	2011–2019
1975–1990	6.781	6.770	6.733	6.811 *
1991–2000	−0.036	6.831	6.756	6.850 *
2001–2010	−0.004	−0.005	6.694	6.784 *
2011–2019	0.166 *	0.180 *	0.182 *	6.509

**Table 4:** The number of dams and sires per number of their foals in each generation interval period.

	Dams Mother of				Sires Father of			
	1975–1990	1991–2000	2001–2010	2011–2019	1975–1990	1991–2000	2001–2010	2011–2019
1 foal	1	7	20	35	2	6	10	24
2 foals	-	6	7	21	1	2	4	10
3 foals	-	1	4	8	-	1	1	-
4 foals	-	-	1	8	-	-	1	3
5 foals	-	-	-	6	-	-	1	1
6 foals	-	-	-	3	-	1	2	1
7 foals	-	-	-	1	-	-	1	1
≥8 foals	-	-	-	-	-	-	-	4
Total	1	14	32	82	3	10	27	41

**Table 5:** The table reports the stallions fathers of foals in 2011–2019, number of their dams and foals, the latter categorized as full sibs. Eight foals born but not having sibs in the period were excluded.

Sire ID	N. Dams	N. Foal	N. Full sibs
A	39	50	22
B	28	46	33
C	14	22	14
D	11	11	0
E	6	7	2
F	5	6	2
G	4	4	0
H	3	4	2
I	3	4	2
J	2	2	0
K	2	2	0
L	2	2	0
M	2	5	5
N	1	2	1
Total (14)	122	167	83

## Data Availability

The data presented in this study are available on request from the corresponding author.

## Supplementary Materials

The following are available online at https://www.mdpi.com/article/10.3390/ani12010098/s1: Table S1: Genetic variability parameters of the related and unrelated LH horse bred in Italy in the period 1975–2019. Table S2: Genetic variability parameters of the unrelated LH horse bred in Italy in the period 1975–2019. Table S3: Genetic variability parameters of the related and unrelated LH bred in Italy in the generational interval periods 1975–1990; 1991–2000; 2001–2010. Each generational interval included all the individuals born in the corresponding years. Figure S1: Variability parameters of the single markers in related and unrelated LH in each generation interval period. Figure S2: Mean values of variability parameters in related and unrelated LH in each generation interval period. Table S4: Level of heterozygosity excess calculated under different mutation models in the unrelated horses on the four generation interval groups and in total population (related and unrelated) in the same four generation intervals [33,34,35]. Observed and expected number of loci with heterozygosity excess and *p*-value of Sign Test; Wilcoxon sign rank test probability value (P); IAM infinite allele model, SMM stepwise mutation model, TPM two-phase model. Figure S3: Expected numbers of loci with heterozygosity excess by Bottleneck software. In the plot “L” shaped distribution of allelic class vs proportion of alleles in related and unrelated LH (periods 1975–1990 and 1991–2000) and unrelated LH (1975–2010 and 2011–2019). Table S5: Genetic variability parameters of LH population bred in Italy compared to parameters reported in other countries [36]. Table S6: Allele frequencies at STR HTG7 in related and unrelated LH in each generational interval periods.
