# Supplementary material for: Genetic Variability Trend of Lusitano Horse Breed Reared in Italy
Source: Animals (Basel). 2022 Jan 1;12(1):98. doi: 10.3390/ani12010098 (PMC8749805; doi:10.3390/ani12010098)
Supplement: Supplementary file 1 [file animals-12-00098-s001.zip › animals-1445919-supplementary.pdf]

Table S1. Genetic variability parameters of the related and unrelated LH horse bred in Italy in the period 1975-2019

Locus	Na	Ae	Ar	Ho	He	N_He	F_{is}	P-val
AHT4	8	4.477	8.000	0.776	0.778	0.777	0.002	0.002
HMS7	7	3.626	6.878	0.740	0.725	0.724	-0.020	0.329
HTG4	7	3.198	6.983	0.716	0.688	0.687	-0.041	0.620
VHL20	9	6.155	9.000	0.815	0.839	0.838	0.028	0.004
AHT5	7	4.180	6.994	0.787	0.762	0.761	-0.033	0.265
ASB2	9	4.447	9.000	0.700	0.776	0.775	0.099	0.001
ASB23	7	4.928	7.000	0.818	0.798	0.797	-0.025	0.240
HMS6	6	2.969	6.000	0.642	0.664	0.663	0.033	0.788
HTG6	8	4.525	7.998	0.720	0.780	0.779	0.077	0.001
HMS2	10	4.109	9.991	0.731	0.758	0.757	0.036	0.261
HMS3	9	6.563	8.880	0.804	0.849	0.848	0.053	0.001
HTG10	9	3.879	8.999	0.711	0.743	0.742	0.044	0.107
HTG7	8	1.928	7.961	0.266	0.482	0.482	0.449	0.001
ASB17	13	4.727	12.970	0.770	0.790	0.788	0.026	0.096
CA425	8	2.991	8.000	0.611	0.667	0.666	0.083	0.001
HMS1	7	2.816	7.000	0.468	0.646	0.645	0.275	0.001
**Mean**	**8.25**	**4.095**	**8.228**	**0.692**	**0.734**	**0.733**	**0.057**	**0.001**
**Total alleles**	**132**							

Na = Observed number of alleles; Ae = Effective number of alleles; Ar= Allelich richness; Ho and He = observed and expected heterozygosity; N_He = Nei's (1973) expected heterozygosity; F_{is} *= inbreeding coefficient; P-val = genetic equilibrium according to Hardy-Weinberg.*

Table S2. Genetic variability parameters of the unrelated LH horse bred in Italy in the period 1975-2019

Locus	Na	Ae	Ar	Ho	He	N_He	F_{is}	P-val
AHT4	8	5.125	7.786	0.745	0.813	0.805	0.085	0.406
HMS7	6	3.114	5.892	0.660	0.686	0.679	0.039	0.490
HTG4	6	3.607	5.883	0.702	0.731	0.723	0.039	**0.942**
VHL20	**9**	6.302	8.882	0.745	0.850	0.841	**0.125**	0.377
AHT5	7	3.922	6.976	0.698	0.754	0.745	0.075	0.791
ASB2	8	4.341	7.819	0.761	0.778	0.770	0.022	0.425
ASB23	7	5.187	6.977	**0.814**	0.817	0.807	0.003	0.870
HMS6	6	2.997	5.894	0.660	0.674	0.666	0.021	0.121
HTG6	7	3.822	6.883	0.766	0.746	0.738	**-0.027**	0.508
HMS2	**9**	4.979	8.739	0.783	0.808	0.799	0.032	0.531
HMS3	8	**6.345**	7.913	0.761	**0.852**	**0.842**	0.108	0.153
HTG10	**9**	3.356	8.819	0.717	0.710	0.702	-0.011	0.587
HTG7	6	**1.588**	5.680	**0.340**	**0.374**	**0.370**	0.091	0.167
ASB17	**9**	4.977	**8.976**	0.721	0.809	0.799	0.109	0.327
CA425	8	3.167	8.000	0.620	0.693	0.684	0.107	**0.033**
HMS1	**5**	2.019	**4.976**	0.465	0.511	0.505	0.090	0.659
**Mean**	**7.37**	**4.053**	**7.255**	**0.684**	**0.725**	**0.717**	**0.056**	**0.423**
**Total alleles**	**118**							

Na = Observed number of alleles; Ae = Effective number of alleles; Ar= Allelich richness; Ho and He = observed and expected heterozygosity; N_He = Nei's (1973) expected heterozygosity; F_{is} *= inbreeding coefficient; P-val = genetic equilibrium according to Hardy-Weinberg.*

Table S3. Genetic variability parameters of the related and unrelated LH bred in Italy in the generational interval periods 1975-1990; 1991-2000; 2001-2010. Each generational interval included all the individuals born in the corresponding years.

Locus	1975-90					1991-00					2001-10					2010-19				
	**Ho**	**He**	**N_He**	**F_{is}**	**P-Val**	**Ho**	**He**	**N_He**	**F_{is}**	**P-Val**	**Ho**	**He**	**N_He**	**F_{is}**	**P-Val**	**Ho**	**He**	**N_He**	**F_{is}**	**P-Val**
**AHT4**	**0.929**	0.808	0.794	**-0.152**	0.525	0.679	0.821	0.813	0.174	**0.001**	0.766	0.787	0.783	0.026	0.339	0.788	0.742	0.740	-0.062	0.180
**HMS7**	0.679	0.700	0.688	0.031	0.725	0.714	0.713	0.707	-0.001	0.607	0.664	0.701	0.698	0.054	0.528	0.798	0.740	0.738	-0.078	0.086
**HTG4**	0.786	0.742	0.729	-0.060	0.584	0.714	0.711	0.705	-0.005	0.886	0.766	0.710	0.706	**-0.080**	0.287	0.679	0.648	0.646	-0.048	**0.602**
**VHL20**	0.857	**0.868**	**0.852**	0.012	0.397	**0.857**	0.841	0.834	-0.019	0.574	**0.851**	**0.833**	0.829	-0.021	0.137	0.777	0.766	0.764	-0.014	0.004
**AHT5**	0.607	0.798	0.784	0.243	0.165	0.782	0.782	0.775	0.000	0.102	0.786	0.753	0.749	-0.044	**0.607**	0.816	0.756	0.754	**-0.080**	0.319
**ASB2**	0.741	0.797	0.782	0.071	0.052	0.643	0.788	0.781	0.185	0.029	0.673	0.825	0.821	0.185	**0.001**	0.725	0.696	0.694	-0.042	0.179
**ASB23**	0.783	0.787	0.770	0.006	0.197	0.792	0.795	0.787	0.004	0.538	0.818	0.812	0.807	-0.008	0.149	**0.829**	0.785	0.783	-0.056	0.174
**HMS6**	0.643	0.636	0.625	-0.010	0.443	0.709	0.675	0.669	**-0.051**	0.616	0.617	**0.633**	**0.630**	0.025	0.514	0.637	0.679	0.677	0.062	0.719
**HTG6**	0.750	0.778	0.764	0.037	0.071	0.784	0.813	0.805	0.036	0.003	0.683	0.827	0.822	0.175	**0.001**	0.715	0.734	0.732	0.026	**0.001**
**HMS2**	0.565	0.841	0.822	0.333	0.003	0.813	0.800	0.792	-0.016	**0.997**	0.747	0.773	0.768	0.034	**0.001**	0.724	0.719	0.717	-0.007	0.406
**HMS3**	0.741	0.860	0.844	0.140	**0.531**	0.696	**0.864**	**0.856**	0.195	0.025	0.832	0.863	**0.859**	0.036	0.004	**0.829**	**0.823**	**0.821**	-0.008	0.287
**HTG10**	0.607	0.690	0.677	0.122	0.140	0.643	0.688	0.682	0.067	0.320	0.717	0.726	0.722	0.012	0.371	0.742	0.767	0.765	0.032	0.431
**HTG7**	**0.333**	**0.586**	**0.575**	0.435	**0.001**	**0.300**	**0.620**	**0.614**	0.519	**0.001**	**0.325**	0.645	0.641	**0.498**	**0.001**	**0.223**	**0.277**	**0.276**	**0.196**	**0.001**
**ASB17**	0.750	0.855	0.837	0.125	0.241	0.800	0.816	0.808	0.020	0.459	0.800	0.804	0.799	0.005	0.458	0.751	0.761	0.759	0.013	0.370
**CA425**	0.714	0.747	0.729	0.045	0.675	0.667	0.746	0.739	0.108	0.002	0.747	0.735	0.730	-0.017	0.057	0.534	0.591	0.589	0.097	0.102
**HMS1**	0.522	0.745	0.729	**0.304**	0.020	0.408	0.753	0.746	**0.461**	**0.001**	0.482	0.716	0.712	0.329	**0.001**	0.472	0.529	0.528	0.110	0.005
**Mean**	**0.688**	**0.765**	**0.750**	**0.102**	**0.002**	**0.688**	**0.764**	**0.757**	**0.101**	**0.001**	**0.705**	**0.759**	**0.755**	**0.072**	**0.001**	**0.690**	**0.688**	**0.687**	**-0.002**	**0.002**

Ho and He = observed and expected heterozygosity respectively; N_He = Nei's (1973) expected heterozygosity; F_{is} = inbreeding coefficient; P-val = genetic equilibrium according to Hardy-Weinberg.

Figure S1. Variability parameters of the single markers in related and unrelated LH in each generation interval period.

A Number of alleles (Na)

Locus	1975-90	1991-00	2001-10	2010-19
AHT4	8	8	6	8
HMS7	6	7	6	6
HTG4	5	6	7	7
VHL20	9	9	9	9
AHT5	7	6	6	6
ASB2	7	9	8	8
ASB23	5	7	7	7
HMS6	6	6	6	5
HTG6	7	7	7	8
HMS2	8	7	7	8
HMS3	8	8	8	9
HTG10	7	8	9	9
HTG7	6	4	5	6
ASB17	11	10	11	11
CA425	7	7	8	8
HMS1	6	6	6	7
Tot	113	115	116	122

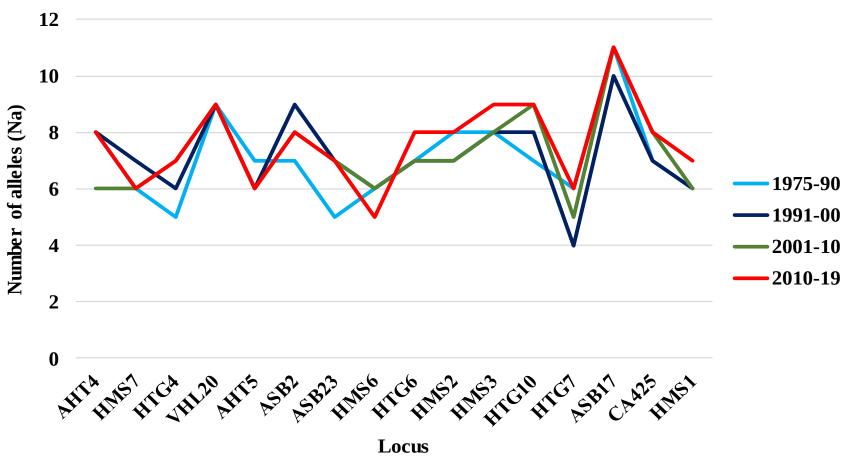

B. Effective number of alleles (Ae)

Locus	1975-90	1991-00	2001-10	2010-19
AHT4	4.85	5.35	4.61	3.84
HMS7	3.20	3.41	3.31	3.82
HTG4	3.69	3.39	3.40	2.83
VHL20	6.76	6.01	5.86	4.24
AHT5	4.63	4.45	3.98	4.07
ASB2	4.59	4.56	5.59	3.27
ASB23	4.35	4.69	5.17	4.61
HMS6	2.67	3.02	2.70	3.09
HTG6	4.24	5.14	5.62	3.74
HMS2	5.63	4.81	4.31	3.53
HMS3	6.40	6.96	7.07	5.57
HTG10	3.10	3.15	3.60	4.25
HTG7	2.35	2.59	2.79	1.38
ASB17	6.13	5.20	4.98	4.15
CA425	3.69	3.82	3.70	2.43
HMS1	3.69	3.93	3.47	2.12
Mean	4.37	4.40	4.39	3.56

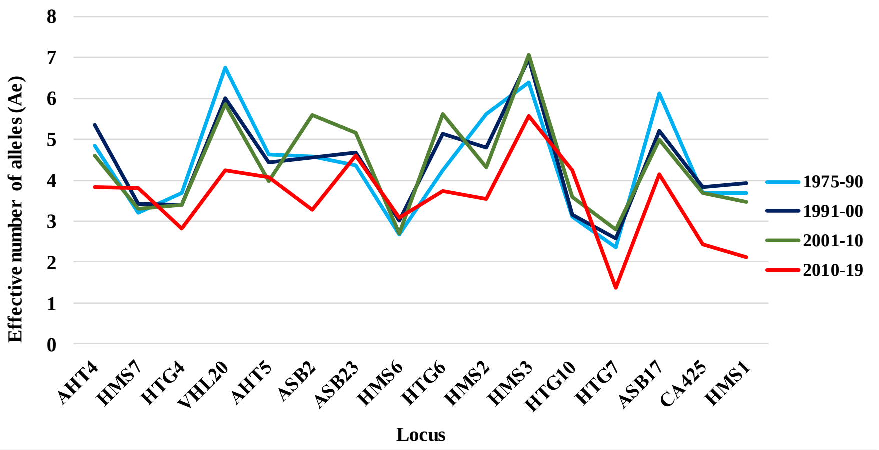

C. Allele richness (Ar)

Locus	1975-90	1991-00	2001-10	2010-19
AHT4	7.44	7.55	5.66	5.99
HMS7	5.72	6.14	5.31	4.89
HTG4	4.75	5.13	4.87	4.51
VHL20	8.88	8.25	8.36	7.11
AHT5	6.69	5.92	5.48	5.50
ASB2	6.97	7.45	7.46	6.41
ASB23	5.00	6.76	6.61	5.71
HMS6	5.74	5.24	5.16	4.72
HTG6	6.73	6.76	6.82	5.38
HMS2	7.83	6.50	6.46	5.85
HMS3	7.98	7.87	7.90	7.18
HTG10	6.86	7.29	6.97	6.32
HTG7	5.51	3.67	4.15	3.79
ASB17	10.48	8.09	8.69	7.96
CA425	7.00	5.95	6.75	4.42
HMS1	5.99	5.42	4.89	4.43
Mean	6.85	6.50	6.35	5.63

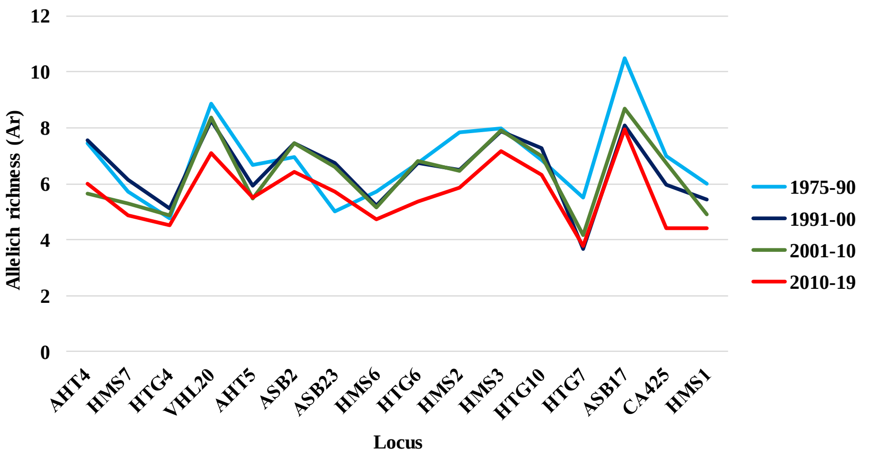

Figure S2. Mean values of variability parameters in related and unrelated LH in each generation interval period.

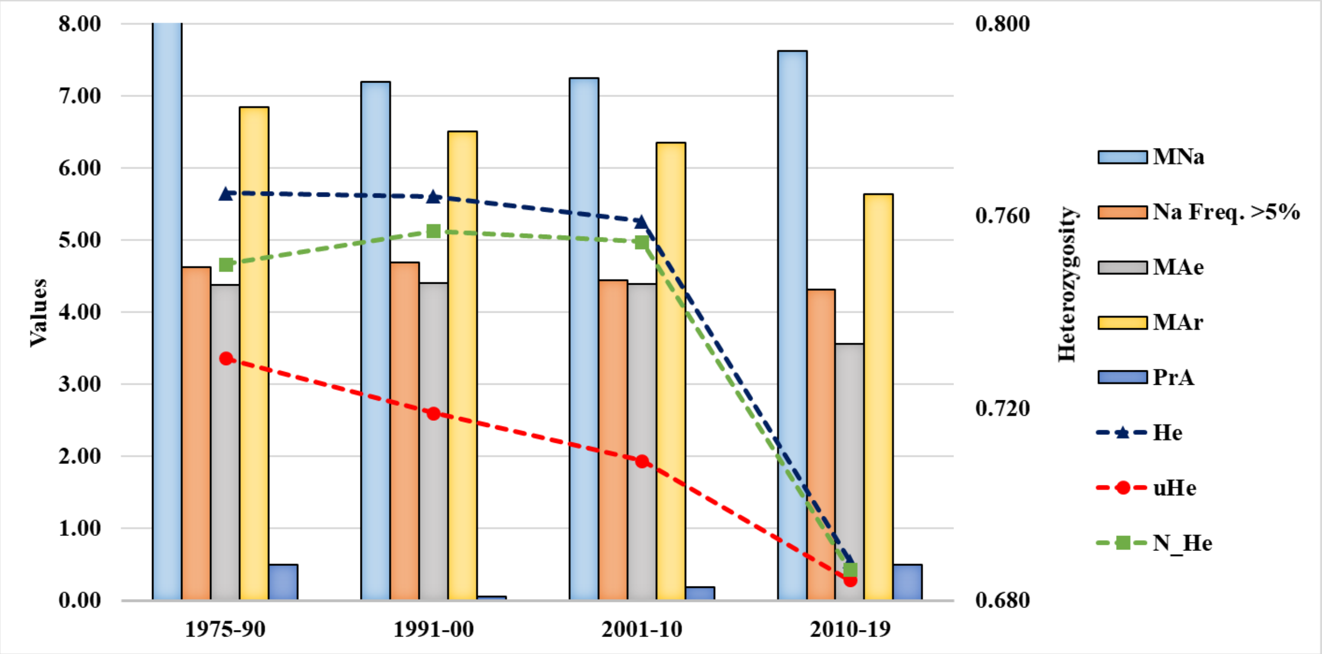

Mean values/period of number of alleles (MNa), number of alleles with a frequency >5% (Na Freq. >5%); Mean number of alleles (MAe), Mean allele richness (MAr), number of Private Alleles (PrA), expected heterozygosity (He), unbiased expected heterozygosity (uHe) and Nei expected heterozygosity (N_He).

TableS4. Level of heterozygosity excess calculated under different mutation models in the total population (related and unrelated) and unrelated horses in different generation intervals. Observed and expected number of loci with heterozygosity excess and P-value of Sign Test; Wilcoxon sign rank test probability value (P); IAM infinite allele model, SMM stepwise mutation model, TPM two-phase model.

LH	period sub-population	Mutation model	Observed loci with heterozygosity excess	Expected loci with heterozygosity excess	P-value Sign Test	Wilcoxon sign rank test (P)
Related and unrelated n.384	1975-90	IAM	13	9.57	0.0629	0.0046*
		SMM	5	9.55	0.0233*	0.9477
		TPM	11	9.49	0.3273	0.0125*
	1991-00	IAM	15	9.32	0.0022*	0.0001*
		SMM	7	9.37	0.1711	0.8510
		TPM	15	9.49	0.0028*	0.0011*
	2001-10	IAM	14	9.34	0.0133*	0.0001*
		SMM	7	9.50	0.1548	0.9281
		TPM	14	9.45	0.0152*	0.0025*
	2011-19	IAM	15	9.38	0.0024*	0.0004*
		SMM	5	9.57	0.0198*	0.9945
		TPM	14	9.39	0.0142*	0.0038*
Unrelated n.47	2001-10	IAM	13	9.51	0.0596	0.0026*
		SMM	9	9.58	0.4768	0.6471
		TPM	13	9.66	0.0689	0.0145*
	2011-19	IAM	13	9.47	0.0572	0.0002*
		SMM	8	9.47	0.3073	0.9035
		TPM	15	9.48	0.0576	0.0042*

**Statistical significance between observed and expected values for heterozygosity excess $p < 0.05$; SM shifted mode, NLS normal L-shaped*

In the four generation interval groups, the Wilcoxon rank test showed that the null hypothesis was accepted under SMM model only, whereas with the Sign test the null hypothesis was accepted under IAM and TPM models in 1975-90 group and under SMM model in 1991-00 and 2001-10 groups. The two unrelated groups showed that the null hypothesis was accepted under the three evolutionary models for Sign test, and under SMM model for the Wilcoxon rank test.

When in a population the majority of loci exhibit an excess of heterozygosity that exceeds the expected at mutation drift equilibrium, such in the present case of LH, a recent bottleneck event can be supposed. To confirm and estimate the excess of heterozygosity the Sign test and the Wilcoxon sign rank tests under different evolutionary models were utilized. The mutation model of evolution followed by microsatellites is still under discussion, so that all the three models available (Infinite allele model -IAM, stepwise mutation model -SMM, and two-phase model of mutation -TPM) were selected for running the Bottleneck program. The most useful markers for bottleneck detection are those evolving under IAM [24, 35, 36]; meanwhile, the TPM is thought to more closely simulate microsatellite mutation and predicts the occurrence of an occasional multiple base-pair repeat [35]. The SMM model predicts all mutations corresponding to the increment or decrement of a single base-pair repeat and is the most conservative model for testing a significant heterozygosity excess caused by bottlenecks [35, 37]. The Mode-shift indicator test discriminates many bottlenecked populations from stable populations and was also utilized as a complementary method to detect potential bottlenecks. The non-bottleneck populations are near mutation-drift equilibrium and are expected to have a large proportion of alleles with low frequency. Loss of rare alleles in bottlenecked populations is detected when one or more of the common allele classes have a higher number of alleles than the rare allele class [25].

Figure S3. Expected number of loci with heterozygosity excess by Bottleneck software. In the plot "L" shaped distribution of allele class vs proportion of alleles in related and unrelated LH (periods 1975-90 and 1991-00) and in unrelated LH (1975-2010 and 2011-19).

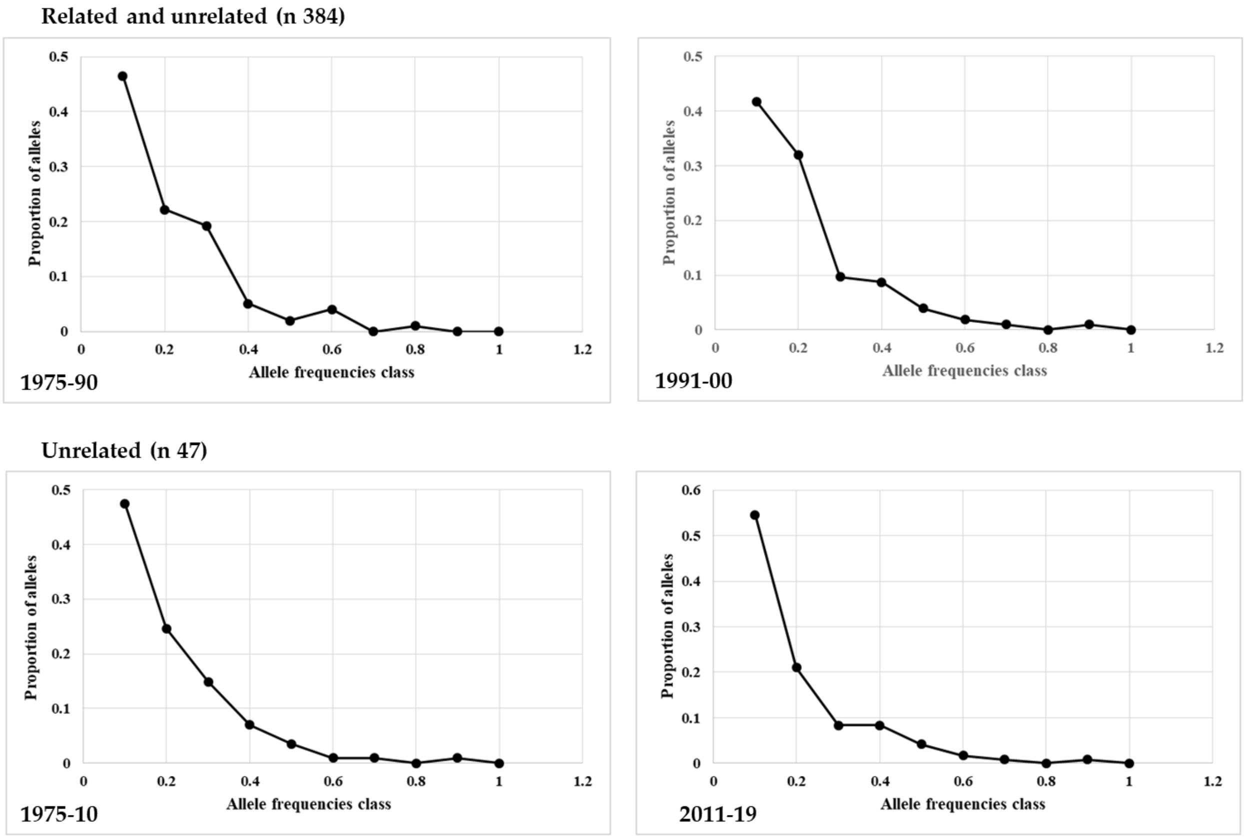

Table S5. Genetic variability parameters of unrelated LH population bred in Italy compared to parameters reported in other countries.

Country	NS	N STR	MNa	He	Ho	F_{is}	Reference
Italy	*47*	*16*	*7.375*	*0.725*	*0.684*	*0.056*	*Present study*
US	40	15	5.33	0.660	0.700	-0.050	[38]
France	50	11	6.70	0.740	0.710	0.040	[9]
Portugal	33	12	6.33	0.721	0.677	-	[5]
Portugal	14,880	9	10.63	0.769	-	0.038	[3]
Brazil	3,097	9	8.88	0.766	-	0.018	[3]
Mexico	458	9	8.25	0.743	-	-0.001	[3]
Spain	373	9	7.75	0.752	-	0.042	[3]

NS = number of samples; N STR = number of microsatellites; MNa = Mean number of alleles; He = expected heterozygosity; Ho = observed heterozygosity; F_{is} *= inbreeding coefficient.*

Table S6. Allele frequencies at STR HTG7 in related and unrelated LH in generational interval periods.

Generational intervals	Alleles							
	K	L	M	N	O	P	Q	R
**1975-90**	0.148	0.037	0.167	0.019	0.611	0.000	0.000	**0.019**
**1991-00**	0.160	0.000	0.300	0.020	0.520	0.000	0.000	0.000
**2001-10**	0.138	0.006	0.344	0.044	0.469	0.000	0.000	0.000
**2010-19**	0.096	0.000	0.023	0.023	0.845	**0.008**	**0.005**	0.000

In bold the private allele exclusively found in the generational interval periods.
